# Pericyte response to ischemic stroke precedes endothelial cell death and blood-brain barrier breakdown

**DOI:** 10.1177/0271678X241261946

**Published:** 2024-07-25

**Authors:** Michaela Roth, Robert Carlsson, Carolina Buizza, Andreas Enström, Gesine Paul

**Affiliations:** 1 Translational Neurology Group, Department of Clinical Science, Wallenberg Neuroscience Center, Lund University, Lund, Sweden; 2 Wallenberg Center for Molecular Medicine, Lund University, Lund, Sweden; 3 Department of Neurology, Scania University Hospital, Lund, Sweden

**Keywords:** Blood-brain barrier, endothelial cells, ischemia, pericytes, stroke

## Abstract

Stroke is one of the leading causes of death and disability, yet the cellular response to the ischemic insult is poorly understood limiting therapeutic options. Brain pericytes are crucial for maintaining blood-brain barrier (BBB) integrity and are known to be one of the first responders to ischemic stroke. The exact timeline of cellular events after stroke, however, remains elusive. Using the permanent middle cerebral artery occlusion stroke model, we established a detailed timeline of microvascular events after experimental stroke. Our results show that pericytes respond already within 1 hour after the ischemic insult. We find that approximately 30% of the pericyte population dies as early as 1 hour after stroke, while ca 50% express markers that indicate activation. A decrease of endothelial tight junctions, signs of endothelial cell death and reduction in blood vessel length are only detected at time points after the initial pericyte response. Consistently, markers of BBB leakage are observed several hours after pericyte cell death and/or vascular detachment. Our results suggest that the pericyte response to stroke occurs early and precedes both the endothelial response and the BBB breakdown. This highlights pericytes as an important target cell type to develop new diagnostic and therapeutic tools.

## Introduction

Ischemic stroke represents the second single most frequent cause of death for people >60 years and the most frequent cause of permanent disability.^1^ Despite this, there is a mismatch between the need for efficient interventions and clinically available treatment options.

Thrombolytic therapy and thrombectomy both aim at reperfusion, but their use is limited to the first hours after stroke onset.^2,3^ A therapy that can be given beyond this time window and that ideally targets different mechanisms of stroke progression is urgently needed.

In ischemic stroke, the acute attenuation of blood flow initiates an ischemic cascade – a sequence of events that includes capillary constriction and ischemic damage to the blood-brain barrier (BBB) followed by the influx of inflammatory cells, ion imbalance, cerebral vascular edema and neuronal death, all of which are associated with a poor prognosis in stroke.^4^ An intervention that targets key cellular components of this ischemic cascade may be crucial to deliver novel stroke treatments. The rational design of such novel therapeutic strategies, however, requires an understanding of the specific cellular and molecular changes in the pathology of stroke progression.

One of the prominent pathological features in stroke that occurs within hours after the ischemic insult is the BBB breakdown,^5^ which leads to the accumulation of blood-derived components and cells within the brain parenchyma which further aggravates inflammation, cellular toxicity and brain damage after an ischemic stroke.^4,6–9^ The BBB, usually a tightly regulated barrier between the blood and the brain parenchyma, is composed of endothelial cells and their linking tight junctions (TJs), astrocytic end feet, the basal lamina, and pericytes.^10–12^

Pericytes are perivascular cells that line the capillaries and have multiple functions in the brain that include the maintenance of the BBB and vessel stabilization. Accordingly, loss of pericytes has been shown to worsen BBB integrity under physiological conditions, as well as during development, aging, and in several neurodegenerative diseases.^13–16^ In stroke, pericytes are now well recognized as major contributors to capillary constriction,^17^ and as key components in regulating the BBB breakdown.^18^ After ischemic stroke, they have been reported to detach from the blood vessels^19,20^ and respond to the hypoxic insult by changing their marker expression.^21–23^ Although targeting the BBB has emerged as an important factor in the development of new therapeutic interventions in the acute phase of stroke,^4,6,24^ most studies analyzing the response of pericytes during the acute phase after stroke are limited to only one timepoint.^17,19,20,25–28^ The exact timeline of the cellular response of BBB-residing cells leading to its breakdown after stroke has not been investigated.

Building upon our findings that provide temporal insights into the alterations of the pericyte transcriptional landscape in a clinically significant model of permanent stroke,^29^ we here investigate the sequence of responses of pericytes and endothelial cells in detail in relation to the breakdown of the BBB, adopting the same stroke model and providing further insight into the temporal response of the microvasculature after ischemic stroke.

We show that pericytes respond within 1 hour after ischemic stroke, whereby they undergo apoptosis or activation. Further, we demonstrate that the pericyte response precedes a decrease of endothelial TJs and endothelial cell death and occurs before a reduction in blood vessel length. Importantly, our data shows that pericyte detachment takes place several hours before the first detection of BBB breakdown. This study pinpoints pericytes as early responders after stroke that react to ischemia in different ways and might constitute an important target to prevent BBB breakdown.

## Material and methods

### Animals

Male wildtype C57bl/6 mice (n = 68) at 8 to 12 weeks of age were used in this study. To visualize Regulator of G-protein signaling 5 (RGS5)^+^ pericytes, *rgs5^+/gfp^* mice (n = 22) were used, allowing to track green fluorescent protein (GFP) expression in pericytes upon activation of the RGS5 promoter.^30^ Animals were housed under standard conditions with access to food and water *ad libitum*. All experimental procedures were approved by the Ethical Committee of Lund University, Lund, Sweden. All experiments were conducted in accordance with the Swedish Board of Agriculture and reported according to the ARRIVE guidelines.

### Permanent Middle cerebral artery occlusion (pMCAO)

To induce a reproducible focal ischemia, the distal part of the left middle cerebral artery (MCA) was permanently occluded as previously described.^31^ In brief, animals were anesthetized with isoflurane and an incision was made between the left lateral part of the orbit and the left ear. The parotid gland and the temporal muscle were moved aside, and a small craniotomy was made with a surgical drill above the anterior distal branch of the MCA. Following exposure, the MCA was permanently occluded by electrocoagulation using an electrosurgical unit (ICC50; Erbe). Marcain (AstraZeneca) was locally applied, and the wound was sutured. Sham animals underwent the same procedure, without occlusion of the MCA.

### Tissue processing

Mice were sacrificed at the following timepoints after pMCAO: 1 h, 3 h, 6 h, 12 h and 24 h. For immunohistochemistry, mice were transcardially perfused with phosphate-buffered saline (PBS) followed by 4% paraformaldehyde (PFA). After the brains were removed, they were placed in 4% PFA for post-fixation overnight, before they were placed in 30% sucrose in PBS for 24 h and then sectioned coronally at 40 µm.

For Western blotting, mice were transcardially perfused with PBS, brains removed and the infarct core and the corresponding area in the contralateral hemisphere dissected. Dissected tissue was frozen immediately on dry ice and stored at −80°C until further processing.

### Immunohistochemistry

For fluorescent immunohistochemistry, the brain sections were washed 3 times in PBS for 5 minutes and then blocked for 30 min in 5% normal donkey or goat serum in 0.25% Triton-X100 in PBS (PBS-TX). Primary antibodies were incubated overnight at room temperature in 3% serum in PBS-TX. For Platelet-derived growth factor receptor ß (PDGFRß) detection, sections were pre-treated with citrate buffer for 20 min at 80°C. The following primary antibodies were used: rabbit anti-PDGFRß (1:200, Cell Signaling, catalog number 3169S), rat anti-CD13 (1:100, AbD Serotec, catalog number mca2183), goat anti-Podocalyxin (1:400, R&D Systems, catalog number AF1556-SP), rabbit anti-neuron-glial antigen 2 (NG2, 1:200, Milipore, catalog number AB5320), rat anti-CD31 (1:400, BD Pharmingen, catalog number 550274), rabbit anti-Fibrinogen (1:400, Abcam, catalog number ab27913), rabbit anti-Ki67 (1:400, Abcam, catalog number ab15580) and chicken anti-GFP (1:5000, Abcam, catalog number ab13970).

After washing, the staining was visualized using species-specific fluorophore-conjugated or biotin-conjugated (Invitrogen) secondary antibodies. Biotinylated secondary antibodies were enhanced with the Vectastain Elite Avidin-biotin complex kit (Vector Laboratories, catalog number PK6100) and revealed using the 3,3-diaminobenzidine (DAB) peroxidase substrate Kit (Vector Laboratories, catalog number SK-4100). Cell death was determined by incubating sections with terminal deoxynucleotidyl transferase-mediated dUTP-X nick end labeling (TUNEL) reaction mix (In Situ Cell death detection Kit, TMR red, Merck, catalog number 12156792910), according to the manufacturer’s instructions.

### Dextran injections

In addition to fibrinogen stainings, BBB breakdown was assessed using intravenous injection of fluorescent-labeled Dextran. Mice were injected with 100 ul of fixable 3kD Dextran-tetramethyl-rhodamine (Thermo Fisher Scientific, catalog number D3308) into the tail vein 30 minutes prior to termination. Perfusion and tissue processing were performed as described above.

### Image processing and cell counting

Brightfield images were taken with an Olympus BX53 light microscope equipped with the digital imaging software CellSense. DAB staining images were obtained at a magnification of 20× and 40×. Fluorescent immunostainings were visualized using a Leica SP8 confocal microscope. To quantify cell numbers, cells were counted in the infarct core on 63x magnified confocal images using 2–3 sections per animal and cell numbers were expressed as numbers/mm^2^. Pericyte coverage was quantified using ImageJ. In brief, images were analyzed with the ImageJ area measurement tool, by which pictures were subjected to threshold processing and producing binary images. The density was then expressed as a percentage of the total area analyzed. Extravasation of fibrinogen and dextran was measured using the endothelial marker CD31 as a counter-staining and analyzed in ImageJ. Figures were assembled in Adobe Illustrator CC 2017 21.0.2.

### Western blot

For protein isolation, brain tissue was cut into small pieces and resuspended in 2% Sodium dodecyl sulfate (SDS) in 50 mM Tris-Cl lysis buffer (pH 7.6) containing 1× Halt Protease inhibitor single-use cocktail (Thermo Fisher Scientific, catalog number 78430) and 1× Halt Phosphatase inhibitor cocktail (Thermo Fisher Scientific, catalog number 78420). Samples were sonicated for 10 seconds at 30% amplitude in a sonicator (Q125 Sonicator, QSonica Sonicators) and centrifuged for 10 minutes at 15000×g at room temperature. Protein concentrations were measured with the Pierce BCA kit (Thermo Fisher Scientific, catalog number 23225). The solubilized lysates were then supplemented to contain 0.1 M dithiothreitol (DTT), 10% glycerol and 0.004% bromophenol blue and heated for 5 minutes at 95°C. Equal total protein amounts (5 µg) were loaded on 4–15% SDS-PAGE TGX-gels (Bio-Rad, catalog number 4561086). The gels were transferred onto nitrocellulose membranes (Bio-Rad, catalog number 170-4159) using the Trans-Blot turbo transfer system from Bio-Rad and incubated for 1 h at room temperature with 5% w/v non-fat dry milk in Tris-buffered saline (TBS) with 0.1% Tween-20 (TBS-T). The membranes were then put in 50 ml tubes and rolled overnight at 4°C with the following primary antibodies diluted in 10 ml 5% milk-TBS-T: rabbit anti-Occludin (1:1000, Abcam, catalog number ab168986) and rabbit anti-Zonula occludens-1 (ZO1, 1:1000, Invitrogen, catalog number 40-2300). After washing, membranes were incubated in 5% milk- TBS-T containing goat anti-rabbit horseradish peroxidase secondary antibody (1:1000, Dako, catalog number P0448) for 1 h, and signal was detected with Clarity, or Clarity Max substrate (Bio-Rad, catalog numbers 1705060 and 1705062, respectively). Images were acquired using ChemiDoc MP system (Bio-Rad) and raw images were analyzed with ImageJ-Fiji. ß-Actin (mouse anti-ß-Actin-Peroxidase, 1:20000, Sigma-Aldrich, catalog number A3854) was used for normalization of gel loading and total protein content. Western blots were repeated twice, quantified and an average was calculated between 2 runs. Quantification of ZO-1 or Occludin protein levels were normalized to ß-Actin. The contralateral hemisphere band intensity was averaged for each time point across the 4 samples and the ipsilateral band intensity from each time point was then divided by the respective averaged contralateral band. Data are expressed as the percentage of the ipsilateral hemisphere compared to the contralateral hemisphere of the same timepoint protein expression levels of Occludin or ZO-1.

### Statistics

Graph Pad Prism versions 7.0, 8.0 and 9.4 were used for statistical analysis of the data. Immunohistochemistry data were analyzed by one-way analysis of variance (ANOVA) followed by Tukey post hoc test. Western blot data were analyzed by two-way ANOVA followed by Sidak post hoc test. Significance was set at p < 0.05 and results are expressed as mean ± SD. Data were checked for statistical outliers by using the Grubbs' test, and for normality by using the Shapiro-Wilk normality test. Blinding was performed to the largest extent possible.

## Results

### Early pericyte death after ischemic stroke

**Figure 1.**
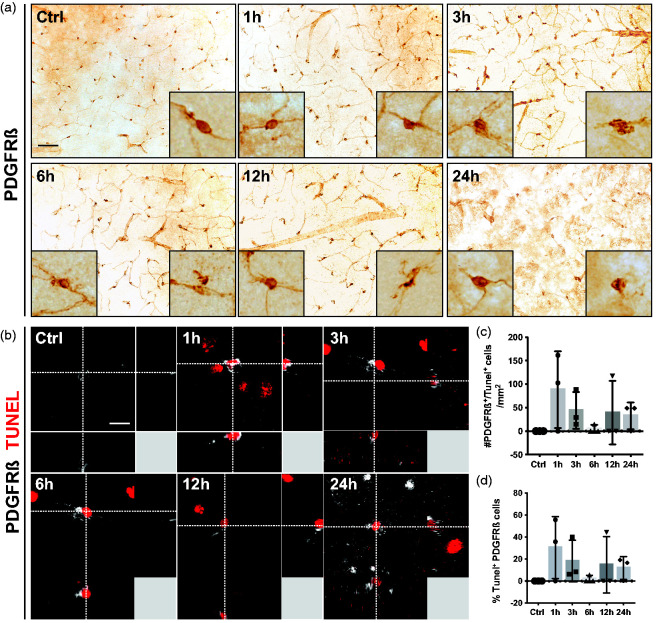
PDGFRß^+^ pericyte cell death occurs after 1 h of stroke. (a) Representative images of PDGFRß staining in the infarct core at different time points after stroke. The boxes in the left lower corner show higher magnification of a typical morphology of perivascular PDGFRß^+^ cells. The boxes in the right lower corner show higher magnification of morphologically abnormal PDGFRß^+^ cells. (b) 3D representation of PDGFRß^+^ pericytes (grey) that are positive for the apoptosis marker TUNEL (red). (c) Quantification of the number of PDGFRß^+^/TUNEL^+^ cells. (d) Quantification of the percentage of PDGFRß^+^ pericytes that are positive for TUNEL. n = 3–4. One-way ANOVA with Tukey’s multiple comparisons. Scale bar 20 µm and 10 µm.

First, we investigated morphological changes in pericytes at 1, 3, 6, 12 and 24 hours after ischemic stroke compared to control conditions using the pericyte marker PDGFRß. Under control conditions, PDGFRß^+^ pericytes had exclusively a round cell soma with extended processes (Figure 1(a)) and similar morphologies were also observed at 1 hour after stroke. From 3 hours after stroke and onwards, PDGFRß^+^ pericytes with irregular cell bodies were detected. In order to investigate whether these morphological changes were associated with cell death, we double-labeled pericytes with the apoptotic marker TUNEL (Figure 1(b)). Under control conditions, no TUNEL^+^ pericytes were observed. However, already 1 hour after ischemic stroke we detected PDGFRß^+^/TUNEL^+^ pericytes (Figure 1(b) to (d)), a number that declined at 6 hours and then increased again at the later timepoints, without however, reaching statistically significant differences between the timepoints (Ctrl: 0.0% ± 0.0%; 1 h: 30.4% ± 28.2%; 3 h: 18.2% ± 18.9%; 6 h: 1.9% ± 3.2%; 12 h: 14.7% ± 25.5%; 24 h: 11.9% ± 10.4%) (Figure 1(d)).

### Early pericyte detachment in stroke is compensated by extension of pericyte processes

**Figure 2.**
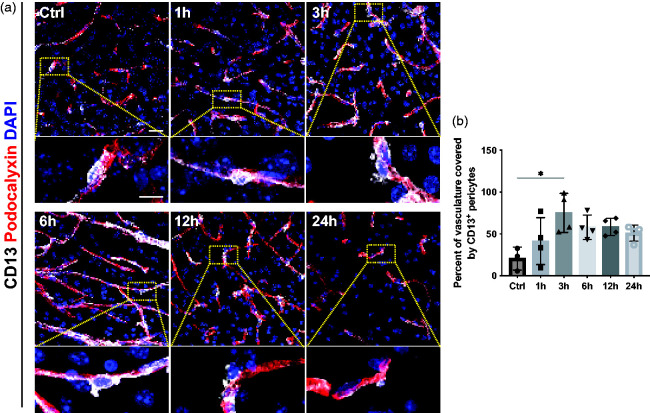
Dynamics of pericyte detachment and changes in pericyte coverage after stroke. (a) Representative confocal images of pericytes (CD13, white) around vasculature (Podocalyxin, red) with nuclear marker DAPI (blue) at different time points after stroke. Higher magnification reveals that after 3 h, CD13^+^ pericytes detach from the vessels. (b) Quantification showing an increase of CD13^+^ pericyte coverage of the vasculature with a maximum at 3 h after stroke, n = 3–4. *p < 0.05. One-way ANOVA with Tukey’s multiple comparisons. Scale bar 20 µm and 10 µm.

As pericyte loss has previously been associated with their early detachment from blood vessels after stroke,^19,20^ we further investigated the localization of pericytes at the vasculature in more detail using the pericyte marker CD13. We confirmed that in sham-operated mice, CD13^+^ pericytes were exclusively found wrapping blood vessels (Figure 2(a)). At 1 hour after stroke, pericyte morphology did not show any detectable changes, in contrast to the following timepoints where CD13^+^ pericytes were seen either detaching from the vessels or extending their processes. The likely compensatory extension of pericyte processes resulted in a higher pericyte coverage of the blood vessels with a maximum observed at 3 hours after stroke (Ctrl: 20.6% ± 13.7%; 1 h: 41.3% ± 28.1%; 3 h: 75.2% ± 23.3%; 6 h: 58.0% ± 14.6%; 12 h: 58.4% ± 10.2%; 24 h: 50.9 ± 9.6%) (Figure 2(b)).

### Pericytes are activated at 1 hour after stroke

**Figure 3.**
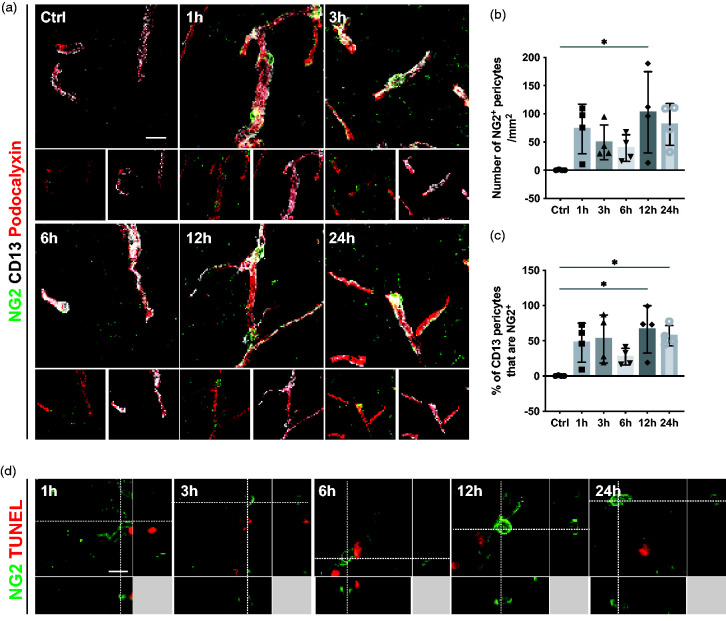
Pericytes are activated and express NG2 from 1 h after stroke. (a) Representative confocal images showing the pericyte activation marker NG2^+^ cells (green), the classic pericyte marker CD13 (grey), and the vasculature (Podocalyxin, red). (b) Quantification of NG2^+^ pericytes at different time points after stroke. (c) Quantification showing the percentage of CD13^+^ pericytes that co-label with NG2. (d) 3D representative confocal images showing that NG2^+^ cells (green) do not co-label with TUNEL (red), n = 4. *p < 0.05. One-way ANOVA followed by Tukey’s multiple comparisons. Scale bar 20 µm and 10 µm.

The response of pericytes is not only restricted to morphological changes but can also be reflected in changes in their marker expression pattern. Pericytes have been shown to increase the expression of NG2 and RGS5 under pathological conditions, which has been described as an indicator of pericyte activation.^32–35^ Therefore, we first investigated the expression pattern of NG2 as a marker of pericyte activation at different timepoints after stroke. In sham-operated mice, we did not detect any perivascular NG2^+^ cells. However, from 1 hour after stroke and for the rest of the selected timepoints, the number of NG2^+^ pericytes was increased (Ctrl: 0.4 ± 0.7 cells/mm^2^; 1 h: 73.1 ± 44.2 cells/mm^2^; 3 h: 49.4 ± 30.7 cells/mm^2^; 6 h: 39.1 ± 23.7 cells/mm^2^; 12 h: 103.0 ± 72.1 cells/mm^2^; 24 h: 81.2 ± 37.0 cells/mm^2^) (Figure 3(a) and (b)). NG2^+^ pericytes showed a typical pericyte morphology and were located around blood vessels (Figure 3(a)). A large part of the CD13^+^ pericyte population co-labeled with NG2 from 1 hour onwards after stroke (Ctrl: 0.3 ± 0.5%; 1 h: 47.2 ± 27.8%; 3 h: 52.5 ± 34.1%; 6 h: 27.3 ± 12.0%; 12 h: 66.1 ± 33.7%; 24 h: 57.1 ± 14.5%) (Figure 3(c)). In addition, we utilized RGS5^+/gfp^ mice, expressing GFP upon RGS5 promotor activity to evaluate RGS5 expression in pericytes.^30^ RGS5 is a commonly used marker for pericytes that has been shown to be upregulated under ischemic conditions and specifically expressed by mural cells in the brain.^22,23^ In these mice, we observed a similar expression pattern showing an increased number of GFP^+^ cells from 1 hour after stroke (Supplementary Figure 1).

We next investigated whether activated and dying pericytes constitute two different populations or whether activation in pericytes was associated with markers of proliferation. At none of the examined timepoints NG2^+^ pericytes co-labeled with TUNEL, indicating that activated pericytes are not undergoing apoptosis (Figure 3(d)). Furthermore, no CD13^+^ pericytes co-labeled with Ki67, suggesting that pericytes do not proliferate within the first 24 hours after ischemic stroke (Supplementary Figure 2).

### Pericyte response precedes tight junction loss after stroke

**Figure 4.**
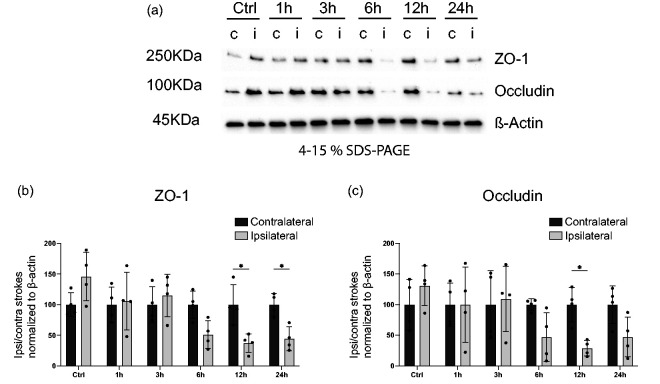
Tight junction loss 12 h after stroke. (a) Western blot (WB) of protein lysates from mouse brain ipsilateral or contralateral hemispheres at indicated time points after stroke or sham surgery. Representative western blots probed for the endothelial TJ proteins ZO-1, Occludin or the loading control ß-Actin. (b–c) Quantification of ZO-1 (b) or Occludin (c) protein levels normalized to ß-Actin. Contralateral hemisphere WB band intensity was averaged for each time point across the 4 samples and ipsilateral band intensity from each time point was then divided by the respective averaged contralateral band. n = 4. *p < 0.05, **p < 0.01. Two-way ANOVA followed by Sidak’s multiple comparisons.

Endothelial cells and their TJs are affected early after stroke.^9,14,27,36^ Therefore, we next investigated the levels of the TJ proteins ZO-1 and Occludin in the stroke-affected (ipsilateral) hemispheres compared to the contralateral hemispheres (Figure 4). As expected, for sham-operated mice, no differences were found in the ZO-1 and Occludin levels between ipsilateral and contralateral hemispheres. ZO-1 protein levels were not different between hemispheres at 1,3 and 6 hours after stroke, but significantly reduced in the ipsilateral hemispheres compared to the contralateral hemispheres at 12 and 24 hours, with a reduction of more than 50%. Occludin levels between hemispheres did not reach a statistically significant difference at 1 and 3 hours. There was a clear trend suggesting Occludin decreases at 6 and 24 hours, however, it did not reach significance (p = 0.87 and p = 0.92), whereas reduction in the Occludin levels at 12 hours was statistically significant (ZO-1 ipsilateral hemispheres: Ctrl: 146.11 ± 39.8%, 1 h: 106.1 ± 47.2%, 3 h: 115.3 ± 34.6%; 6 h: 51.2 ± 22.4%, 12 h: 37.2 ± 15.3%, 24 h: 44.3 ± 19.6%; Occludin ipsilateral hemispheres: Ctrl: 130.8 ± 32.5%, 1 h: 99.9 ± 61.3%, 3 h: 109.4 ± 53.1%; 6 h: 46.8 ± 40.0%, 12 h: 28.8 ± 12.7%, 24 h: 47.3 ± 32.4%) (Figure 4(a) to (c)).

### Endothelial cell death is first detectable at 12 hours and a reduction in vessel length is not observed before 24 hours after stroke

**Figure 5.**
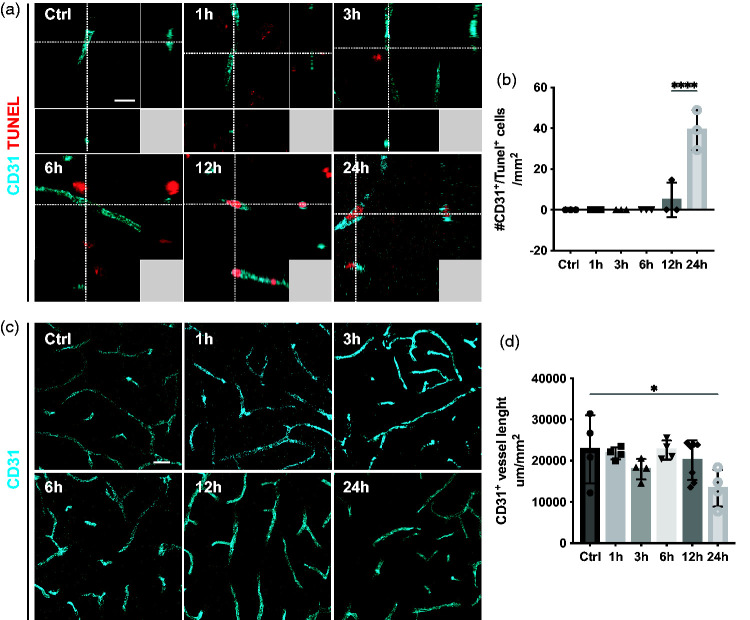
Delayed endothelial cell death and decrease in vessel length. (a) 3D representative confocal images showing that endothelial cells (CD31, cyan) are TUNEL^+^ (red) after 12 h. (b) Quantification of CD31^+^/TUNEL^+^ cells at different time points after stroke. (c) Representative confocal images of vasculature (CD31, cyan) at different time points after stroke. (d) Quantification of CD31^+^ vessel length showing a decrease in total vessel length after 24 h, n = 3–4. *p < 0.05, ****p < 0.0001 towards all other groups. One-way ANOVA with Tukey’s multiple comparisons. Scale bar 10 µm and 20 µm.

To compare the timeline of endothelial and pericyte cell death along with vascular density after ischemic stroke, we next investigated endothelial cell death. Interestingly, we did not detect any CD31^+^ endothelial cells that co-labeled with TUNEL within the first 6 hours after stroke. After 12 hours, we observed a few CD31^+^/TUNEL^+^ endothelial cells, but their number significantly increased at 24 hours compared to controls (Ctrl: 0.0 ± 0.0 cells/mm^2^; 1 h: 0.0 ± 0.0 cells/mm^2^; 3 h: 0.0 ± 0.0 cells/mm^2^; 6 h: 0.0 ± 0.0 cells/mm^2^; 12 h: 4.9 ± 8.5 cells/mm^2^; 24 h: 39.1 ± 9.8 cells/mm^2^) (Figure 5(a) and (b0). Analysis of the vasculature further revealed that the total vessel length decreased significantly at 24 hours after stroke (Ctrl: 22799 ± 8261 µm; 1 h: 21789 ± 1450 µm; 3 h: 17926 ± 2488 µm; 6 h: 22604 ± 2317 µm; 12 h: 20127 ± 4817 µm; 24 h: 13336 ± 4481 µm) (Figure 5(c) and (d)).

### Blood-brain barrier breakdown is detectable at 12 hours after stroke

**Figure 6.**
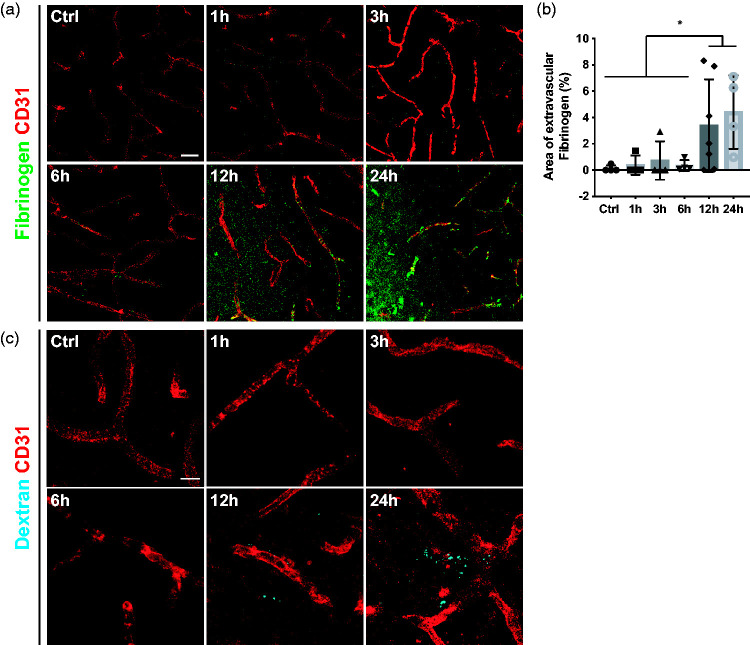
Substantial leakage at 12 h after stroke. (a) Representative confocal images of endothelial cell marker CD31 (red) and endogenous fibrinogen (green) showing increased fibrinogen accumulation after 12 h. (b) Quantification of extravascular Fibrinogen at different time points after stroke. (c) Confocal images of intravenously injected Dextran (cyan) and the endothelial marker CD31 (red) showing that vascular leakage occurred after 12 h, n = 4–7. *p < 0.05. One-way ANOVA with Tukey’s multiple comparisons. Scale bar 20 µm and 10 µm.

Pericytes are an integral part of the BBB, and their loss has been shown to aggravate the BBB breakdown after stroke.^37^ Thus, we investigated at what timepoint vascular leakage was detectable in relation to the pericyte and endothelial cell response by quantifying the accumulation of endogenous fibrinogen in the parenchyma. As expected, in sham-operated mice, there was no extravascular fibrinogen deposition (Figure 6(a) and (b)). Similarly, 1, 3 and 6 hours after stroke, we did not detect fibrinogen leakage in the brain parenchyma. On the other hand, at 12 and 24 hours after ischemic stroke, extravascular fibrinogen was significantly increased (Ctrl: 0.1 ± 0.2%; 1 h: 0.4 ± 0.7%; 3 h: 0.7 ± 1.5%; 6 h: 0.3 ± 0.4%; 12 h: 3.4 ± 3.5%; 24 h: 4.4 ± 2.8%) (Figure 6(a) and (b)).

As fibrinogen is a large size molecule (around 340 kDa), we next investigated if there was an earlier, more subtle leakage by using a small molecular tracer. For this, we injected the fluorescent-labeled 3 kDa Dextran tracer and investigated its penetrance into the brain parenchyma. Similar to the endogenous fibrinogen leakage, extravascular dextran was only observed after 12 hours after ischemic stroke (Figure 6(c)).

## Discussion

Preserving the integrity of the BBB after ischemic stroke is increasingly recognized as a potential strategy to alleviate the pathological progression of the ischemic injury.^18,24,38,39^ Even though pericytes are now widely acknowledged as key players in neurovascular pathology, studies examining their response after acute ischemic stroke are still sparse and mostly limited to one or a few time points.^17,19,20,25–28^ Recently, we have adopted single-cell sequencing to profile the pericytes post-ischemic responses at 1, 12- and 24-hours post-stroke.^29^ In the current study we now investigate a more detailed timeline of different vascular events within the acute phase and with the aid of other techniques, such as immunohistochemistry and WB. By assessing the temporal dynamics of the BBB breakdown at 1, 3, 6, 12 and 24 hours after a permanent middle cerebral artery occlusion stroke model, we observe that already within the first hour pericytes exhibit two distinct behaviors: some undergo apoptosis, consistent with prior research,^17^ while others become activated expressing NG2 and RGS5, and evading apoptotic processes.

**Figure 7.**
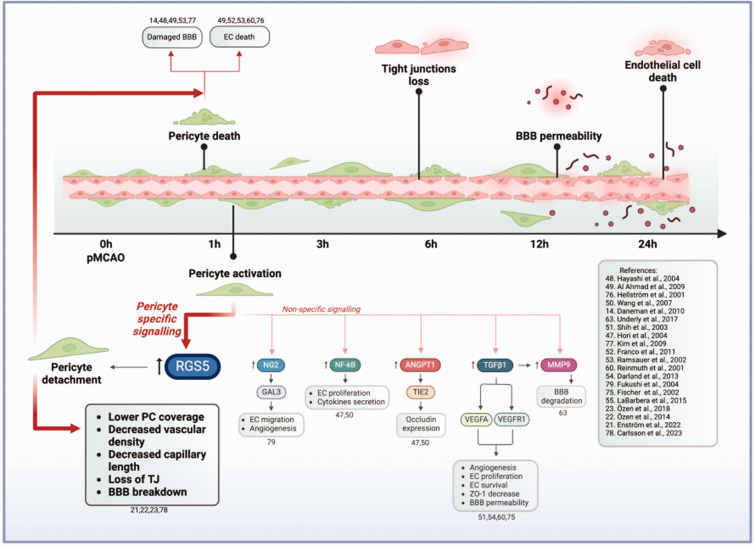
Schematics on the timeline of early microvascular events after pMCAO, based on the current study, and possible underlying pericytes signaling mechanisms, based on available literature. ↑ indicates an increased expression in the markers. 1 hour after pMCAO, pericytes either undergo apoptosis or become activated. Based on existing literature including studies both *in vivo* and *in vitro* mimicking stroke in co-culture with endothelial cells or conditioned media models, pericytes have been reported to express or upregulate markers such as RGS5, NG2, NF-κB, ANGPT1, TGFβ1, MMP9. These molecules have been linked to subsequent effects on BBB disruption and endothelial cells survival. Among those, RGS5 is the only molecule expressed exclusively by pericytes in the brain. In hypoxic environments, RGS5 regulates pericyte detachment from the vascular wall and migration into the brain parenchyma, leading to decreased vessel coverage, lower vascular density, loss of TJs and BBB breakdown. Since pericytes play an essential role in the formation and maintenance of the TJ both *in vivo* and *in vitro*, early pericyte death can also be linked to the decrease in TJs. pMCAO = permanent occlusion of the middle cerebral artery; RGS5 = regulator of G-protein signalling 5; NG2 = neural-glia antigen 2; NF-κB = nuclear factor kappa-B; ANGPT1 = angiopoietin-1; TGFβ1 = transforming growth factor beta; MMP9 = matrix metalloproteinases 9^21–23,47–55,60,63,75–79^*. Created with BioRender.com, 05 April 2024.*

Within 3 hours post-stroke, pericytes undergo morphological changes and show signs of detachment from the blood vessel wall, corroborating previous studies.^19,20^ Interestingly, not all the detaching pericytes undergo apoptosis. We and others have previously shown that detached pericytes can migrate into the parenchyma and change their phenotype, possibly playing a role in inflammation and scar formation after ischemic stroke.^22,40^ As a possible underlying mechanism of detachment, we have demonstrated in other studies that pericyte recruitment and retention to the vascular wall is counteracted by the early expression and protein stabilization of RGS5 in pericytes in hypoxia, whereby RSG5 expression desensitizes pericytes to endothelial platelet-derived growth factor-BB (PDGF-BB) chemotactic cues (Figure 7).^21,23^ RGS5 is exclusively expressed by pericytes in the brain.^29,41,42^ Hypoxic conditions rapidly stabilize the RGS5 protein, while RGS5 is rapidly degraded in normoxia.^21^ We have previously demonstrated that RGS5 is linked to the migration of pericytes away from the capillaries, which causes subsequent BBB leakage. Overall, RGS5 early expression in pericytes might contribute to the subsequent endothelial cell death and TJ loss after ischemic stroke.^21,23^

Consistently, loss of RGS5 in RGS5 knockout mice results in increased perivascular pericyte numbers and better pericyte coverage of the blood vessels, preserved tight junctions and significantly less BBB leakage, and improved capillary density and length.^23^ Those studies show that the hypoxic stimulus activates RGS5 in pericytes leading to other downstream events which can be prevented by modulating the response of pericytes to hypoxia.

We observe a decrease in the TJ proteins ZO-1 and Occludin beginning between 6 and 12 hours post-stroke, followed by endothelial cell death at 12 and 24 hours, and a reduction in vessel length at 24 hours, potentially as a direct consequence of endothelial cell death.^43^ This delayed endothelial cell death aligns with prior studies indicating limited endothelial cell loss within the first 24 hours following stroke.^17,44,45^

Based on the timeline of events we propose that pericyte death, detachment and activation precedes TJ loss and endothelial cell death. Pericytes loss has been suggested in the literature to be a major contributor to the decrease in TJs^14,39,46,47^ and BBB breakdown after stroke.^13,39^ We have shown that deletion of RGS5 in pericytes in stroke protects TJs.^23^ Interestingly, Hayashi et al.^48^ observed the tightest barrier in an endothelial/pericyte co-culture model even in hypoxic conditions, but only when the cells were in contact. Pericytes are also well known for preserving endothelial cell survival both in physiological and pathological conditions mimicking stroke.^47–55^ Several signaling pathways have been reported to play a role in this interaction,^52,56–59^ including Transforming Growth Factor Beta 1 (TGFβ1) – Vascular Endothelial Growth Factor Receptor 1,^51^ Vascular Endothelial Growth Factor A,^52,60^ Nuclear factor NF-kappa-B^55,61^, and Angiopoietin-1 (Figure 7).^47,50^

At the same time, the activated phenotype of the pericytes may promote BBB opening. During angiogenesis, pericytes degrade the basement membrane, promote vessel sprouting and endothelial cell migration.^62^ In pathological conditions, these features might result in a pathological BBB opening and leakage. In line with this, previous studies showed that during ischemia pericytes secrete TGFβ1 and matrix metalloproteinases-9 (MMP9), possibly as a fast angiogenic response to hypoxia.^63^ In addition, inflammatory conditions, such as in ischemic stroke, could induce the pericytes to produce proinflammatory cytokines, chemokines and other pro-inflammatory factors,^29,64–67^ which could further impair the cellular microenvironment and disrupt the BBB.

In conclusion, we observe a rapid, dynamic, and differential response of pericytes after stroke that precedes endothelial cell death and BBB leakage. Further studies are required to define the specific mechanisms even better with which pericytes disrupt the BBB integrity in stroke, however, the accumulating evidence is supporting that modulation of the early response of pericytes may prevent or moderate further downstream injuries associated with BBB damage.

One limitation of our study is the use of exclusively male mice, motivated by the aim to control for variables like sex, age, and genetic background. However, growing evidence suggest that sexual dimorphism has an impact on both healthy^68,69^ and pathological neurovascular functions, such as pathological brain lipid metabolism^70^ and neurovascular disorders.^71^ Importantly, sexual dimorphism also affects prevalence and outcome of ischemic stroke.^72–74^ Considering the recognized variations in cerebrovascular functions between males and females in health and disease, future studies that include both female and male mice are highly warranted and may provide valuable insights into sex-specific differences in BBB responses after ischemic stroke.

## Supplemental Material

sj-pdf-1-jcb-10.1177_0271678X241261946 - Supplemental material for Pericyte response to ischemic stroke precedes endothelial cell death and blood-brain barrier breakdown Supplemental material, sj-pdf-1-jcb-10.1177_0271678X241261946 for Pericyte response to ischemic stroke precedes endothelial cell death and blood-brain barrier breakdown by Michaela Roth, Robert Carlsson, Carolina Buizza, Andreas Enström and Gesine Paul in Journal of Cerebral Blood Flow & Metabolism

sj-pdf-2-jcb-10.1177_0271678X241261946 - Supplemental material for Pericyte response to ischemic stroke precedes endothelial cell death and blood-brain barrier breakdown Supplemental material, sj-pdf-2-jcb-10.1177_0271678X241261946 for Pericyte response to ischemic stroke precedes endothelial cell death and blood-brain barrier breakdown by Michaela Roth, Robert Carlsson, Carolina Buizza, Andreas Enström and Gesine Paul in Journal of Cerebral Blood Flow & Metabolism

sj-pdf-3-jcb-10.1177_0271678X241261946 - Supplemental material for Pericyte response to ischemic stroke precedes endothelial cell death and blood-brain barrier breakdown Supplemental material, sj-pdf-3-jcb-10.1177_0271678X241261946 for Pericyte response to ischemic stroke precedes endothelial cell death and blood-brain barrier breakdown by Michaela Roth, Robert Carlsson, Carolina Buizza, Andreas Enström and Gesine Paul in Journal of Cerebral Blood Flow & Metabolism
