# Supplementary figures and images for: Pericyte response to ischemic stroke precedes endothelial cell death and blood-brain barrier breakdown
Source: J Cereb Blood Flow Metab. 2024 Jul 25;45(4):617–29. doi: 10.1177/0271678X241261946 (PMC11571979; doi:10.1177/0271678X241261946)

# Supplementary Figure 1

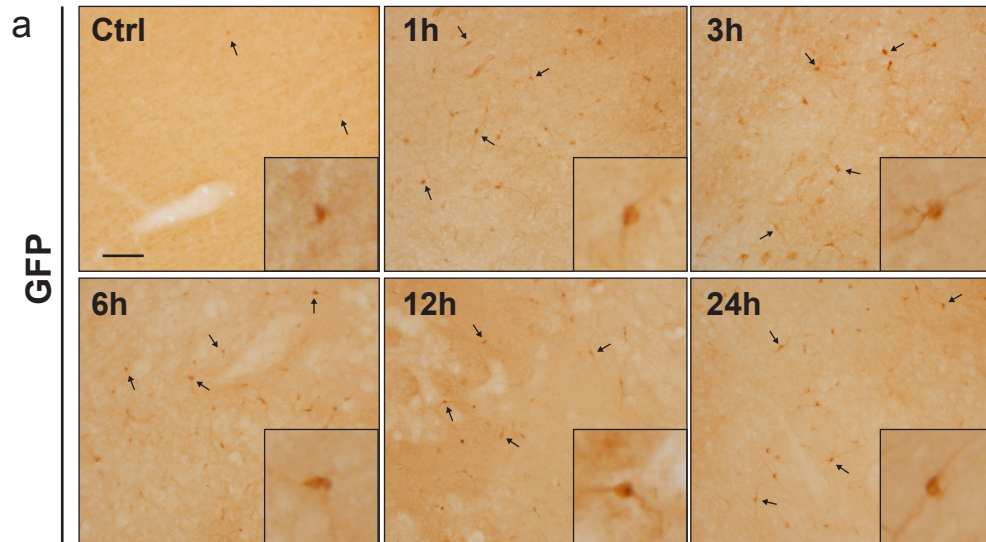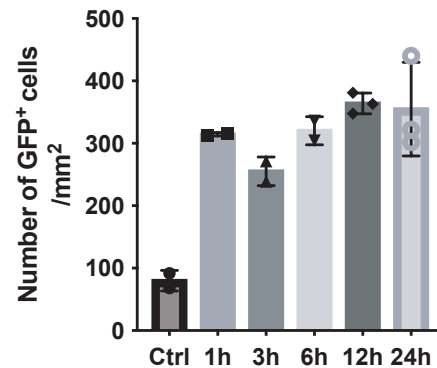

Supplement: sj-pdf-1-jcb-10.1177_0271678X241261946 - Supplemental material for Pericyte response to ischemic stroke precedes endothelial cell death and blood-brain barrier breakdown [file sj-pdf-1-jcb-10.1177_0271678X241261946.pdf]

# Supplementary Figure 2

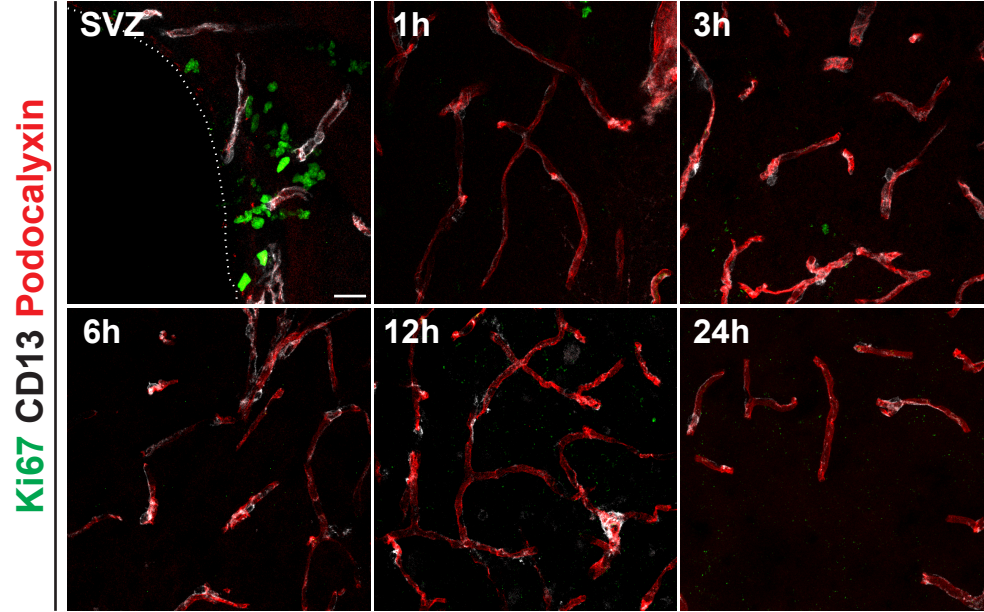

Supplement: sj-pdf-2-jcb-10.1177_0271678X241261946 - Supplemental material for Pericyte response to ischemic stroke precedes endothelial cell death and blood-brain barrier breakdown [file sj-pdf-2-jcb-10.1177_0271678X241261946.pdf]
