# Supplementary material for: Pericyte response to ischemic stroke precedes endothelial cell death and blood-brain barrier breakdown
Source: J Cereb Blood Flow Metab. 2024 Jul 25;45(4):617–29. doi: 10.1177/0271678X241261946 (PMC11571979; doi:10.1177/0271678X241261946)
Supplement: sj-pdf-3-jcb-10.1177_0271678X241261946 - Supplemental material for Pericyte response to ischemic stroke precedes endothelial cell death and blood-brain barrier breakdown [file sj-pdf-3-jcb-10.1177_0271678X241261946.pdf]

*Supplementary Figure 1: The number of pericytes expressing RGS5 is increased 1h after stroke.*

**a.** Representative images of RGS5-GFP$^+$ pericytes at different time points after stroke. The arrows indicate examples of GFP$^+$ cells. The boxes in the lower right corner show higher magnification of RGS5-GFP$^+$ pericytes. **b.** Quantification showing an increase in RGS5-GFP$^+$ cells 1h after stroke, n=2-3. Scale bar 20 µm.

*Supplementary Figure 2: Pericytes do not proliferate within the first 24h after stroke.*

**a.** Representative images of CD13$^+$ pericytes (white) and the proliferation marker Ki67 (green) with the vasculature (red), showing that at none of the time points CD13$^+$ pericytes co-label with Ki67. The subventricular zone (SVZ) in a sham-operated mouse served as a positive control for Ki76$^+$ cells. The dotted line in SVZ image indicates the outline of the tissue, n=3, Scale bar 20 µm.
